# Transdiagnostic Mechanisms of Mental Health During the COVID-19 Pandemic on Adults and Families in Germany: Study Protocol of a Cross-Sectional and 1-Year Longitudinal Study

**DOI:** 10.3389/fpsyg.2021.720104

**Published:** 2021-12-22

**Authors:** Jana Volkert, Svenja Taubner, Anna Berning, Laura Kling, Hannah Wießner, Anna K. Georg, Julia Holl

**Affiliations:** ^1^MSB Medical School Berlin, Berlin, Germany; ^2^Institute for Psychosocial Prevention, Heidelberg University, Heidelberg, Germany

**Keywords:** pandemic (COVID-19), crisis, trauma, reflective functioning, mentalizing, personality functioning, emotion regulation, family

## Abstract

**Background:** Since the outbreak of COVID-19 pandemic, psychological distress is increased. Transdiagnostic mechanisms, including trauma, personality functioning, mentalizing and emotion regulation are considered relevant to the development and maintenance of mental health problems and therefore may play a role in individuals’ reactions to the pandemic.

**Aim:** To identify moderating and mediating factors associated with pandemic-related distress and mental health problems in adults and families, we aim to investigate the interactions of interpersonal trauma (childhood trauma and domestic violence), psychological capacities (personality functioning, mentalizing and emotion regulation) and pandemic-related adversity on psychological distress during the COVID-19 pandemic. Furthermore, we aim to investigate behavioral and cognitive consequences of the pandemic (e.g., media consumption, vaccination status, conspiracy beliefs).

**Methods:** Using an online-based cross-sectional and longitudinal design, we will investigate a sample of adult participants recruited *via* online platforms in German-speaking countries over the course of 1 year with four measurements points *via* self-report instruments (personality functioning: PID5BF +; mentalizing: MentS, PRFQ; emotion regulation: DERS-SF; mental health problems: PHQ-9, GAD-7; a composite pandemic-related stress score). Structural equation and multi-level modeling will be performed for data analyses.

**Implications:** This study will provide data on the moderating and mediating effects of trauma, personality functioning and mentalizing during the pandemic in a large community sample, particularly on vulnerable groups like families. Identifying transdiagnostic mechanisms of psychopathology in the course of a pandemic crisis may provide valuable insight for the development of pre- and intervention measures for potential psychological distress during and post the pandemic.

## Introduction

The current COVID-19 pandemic has a massive impact on all people’s life’s including individual, family related, societal, economic and financial hardship, social and cultural deprivation, and educational disadvantages (e.g., Kujawa et al., 2020).

A number of studies examined the psychological impact of the COVID-19 pandemic showing that psychological distress is generally increased across populations worldwide (e.g., Salari et al., 2020; Wu et al., 2020). In Europe, several studies have shown an increase in psychological distress related to the pandemic (Pierce et al., 2020; Rodríguez-Rey et al., 2020; Rossi et al., 2020). Studies from Germany, in particular, are also in line with international findings, showing for example significantly higher levels of depression, anxiety and increased psychological distress in the general population during the pandemic and specifically during lockdown phases (Bäuerle et al., 2020; Benke et al., 2020; Petzold et al., 2020). Studies have found a number of factors associated with psychological distress during the pandemic, namely being in quarantine, loneliness, worries about infections or death of important others, unemployment and housework, as well as social and financial consequences of the pandemic (Benke et al., 2020; Chandola et al., 2020; Rodríguez-Rey et al., 2020; Rossi et al., 2020).

Vulnerable populations, like families with young children are particularly affected during the pandemic (Pierce et al., 2020). Factors that have been identified to be associated with families’ psychological distress are their life circumstances (for example work- and learning spaces at home) (Pierce et al., 2020), financial anxiety and lack of social support. While the burden is higher in those families with younger children and those with two or more children (Mazza et al., 2020; Li et al., 2020), the impact on the mental health of young children, i.e., infants and toddlers, has been less investigated. Furthermore, several studies found an increase in intimate partner violence and child maltreatment since the COVID-19 pandemic (e.g., Brown et al., 2020; Jetelina et al., 2021). Decreased service utilization, other COVID-19 related stressful events, and increased parental psychological symptoms were also related to higher child abuse potential (Brown et al., 2020; Lawson et al., 2020). However, so far little is known about psychological risk and protective factors of families, which may amplify or buffer the potential effect from COVID-19 related adversities and parental distress on risk of child abuse.

Drawing on existing transdiagnostic models of childhood trauma and psychopathology (McLaughlin et al., 2020), and mentalizing (Luyten et al., 2020), we aim to expand this to the understanding of the psychosocial impact of the pandemic. Thereby, we aim to investigate the relationship of childhood trauma, psychological capacities including personality functioning, emotional awareness and regulation, and mentalizing abilities on the psychosocial impact of the pandemic. Social risk factors like younger age, female gender or unemployment, in addition to families with young children, have been shown to play an important role in experiencing increased psychological distress during the pandemic (Horesh and Brown, 2020; Xiong et al., 2020). Fewer studies have focused on factors such as a history of traumatic experiences or personality factors (e.g., Fernández et al., 2020). In the following, we briefly outline the evidence based on these hypothesized risk and protective factors with regard to mental health problems and summarize current findings on the pandemic impact:

A history of interpersonal childhood trauma (for example physical, sexual, and emotional abuse or neglect) is associated with a higher risk for a psychopathological development later in life (Felitti et al., 1998; Mandelli et al., 2015). With regard to pandemic-related impacts on mental health, Guo et al. (2020) found that adolescents with pre-pandemic traumatic experiences were associated with increased anxiety during the pandemic, and Kim et al. (2020) found a moderating effect of early childhood trauma on the association between the perceived risk from COVID-19 and depression. Hence, experiences of childhood trauma may be understood as a vulnerability factor for psychopathology during the pandemic.

Personality functioning, conceptualized as basic psychological capacities of a person have an understanding and regulation abilities of the self of relationships with others, is considered a key factor to develop and maintain mental health (Kernberg and Caligor, 2005; Bender et al., 2011). With regard to the pandemic, a disposition toward internalizing personality traits (including negative affectivity, detachment, closed-mindedness and psychoticism), were associated with increased levels of depression, anxiety and stress during the pandemic (Mazza et al., 2020; Biondi et al., 2021) and elevated levels of negative affectivity were found to contribute to lower resilience and reduced subjective well-being (Kocjan et al., 2021).

Mentalizing - the basic human capacity to conceive oneself and others as intentional beings whose actions are guided by feelings, thoughts, desires, attitudes, and goals (Fonagy et al., 2002) - is implicated in a number of mental health problems and disorders (Luyten et al., 2020) and can be understood as an adaptive psychological mechanism for coping with pandemic-related stressors. In a similar vein, parents’ ability to mentalize their child may be a protective factor against the adverse effects of pandemic-related stress on the child because parents are able to co-regulate their child more effectively according to its needs (Zeegers et al., 2017). So far, very few empirical studies have investigated pandemic-related effects on mentalizing abilities (Lassri and Desatnik, 2020; Ventura Wurman et al., 2020) and none of the studies focused on mentalizing as a risk or protective factor in the general population or families.

Furthermore, an individual’s ability to experience and regulate emotions is also considered highly relevant to develop and maintain mental health (e.g., Aldao et al., 2010; Hu et al., 2014). According to Gratz and Roemer (2004) the concept of emotional (dys-)regulation comprises (a) the ability to identify and understand emotions, (b) the acceptance of emotions, (c) the potential to exhibit goal-directed behavior and control impulsive behavior even while exposed to negative emotions or stress, and (d) the ability to flexibly use situationally appropriate emotion regulation strategies to achieve individual goals (Kaufman et al., 2016). A recent study examining emotional dysregulation during the pandemic found that having survived a Covid-19 infection was associated with a greater likelihood of psychological distress, and this in turn was associated with greater emotional dysregulation as well as elevated levels of depressive mood (Janiri et al., 2020). Moreover, the concept may be of particular relevance to family’s mental health during the pandemic, as parents’ emotional dysregulation problems have been shown to be related to children’s stress reactions (Shorer and Leibovich, 2020). Young children of parents with deficits in emotion regulation may be at a heightened risk during the pandemic to experience less adaptive co-regulation and thus may show more mental health problems (e.g., regulatory symptoms).

According to transdiagnostic models of the development of mental health problems, interactions between interpersonal trauma, psychological capacities and other factors exist and are to some extend supported by empirical data. For example, Abdi and Pak (2019) found that the effect of maladaptive personality traits and low personality functioning on mental health problems was mediated by emotion dysregulation. Moreover, emotion dysregulation (Cloitre et al., 2019) as well as mentalizing (Huang et al., 2020) are found to serve as mediating factors in the relationship between childhood trauma and psychopathology. These findings indicate the extent to which psychological capacities can have a mediating influence on a psychopathological development against the background of traumatic experiences in early childhood.

Furthermore, the behavioral and cognitive consequences of the pandemic such as increasing media consumption, authoritarianism, conspiracy beliefs, vaccination status and attitude, and posttraumatic growth need to be considered, if we want to advance our understanding about maintaining and losing mental health in a global crisis. Recent studies have shown a new rise in people’s vulnerability to conspiracy beliefs during the pandemic (Šrol et al., 2020; Leibovitz et al., 2021; Oleksy et al., 2021). Dyrendal et al. (2021) identified right-wing authoritarianism within their analysis as one predictor of conspiracy theories. Hartman et al. (2020) also showed a correlation between COVID-19-specific conspiracy beliefs (i.e., that the virus originated on a Chinese laboratory) and a political attitude toward right-wing authoritarianism. These results are also supported by Prichard and Christman (2020), who found that authoritarianism was not only a predictor of less concern about the COVID-19 virus and low compliance, but also associated with the belief that the virus had been produced in China. Moreover, this COVID-19 pandemic may also be regarded an infodemic, in which the overabundance of information, individuals’ increased media consumption and deliberate attempts to disseminate wrong information effects people’s psychological capacities and mental health (Cellini et al., 2020; Valdez et al., 2020; World Health Organization [WHO], 2020, September 23). At the same time, people may also be able to cope with the crisis in a positive way and develop posttraumatic growth as a result of the pandemic (e.g., Vazquez et al., 2021).

### Rationale and Aim of This Study

Based on the research outlined above, we aim to investigate a multi-factorial and transdiagnostic model of risk and protective factors associated with pandemic-related distress and psychological impairments in adults and families in the general German-speaking population during the COVID-19 pandemic in a cross-sectional and 1-year longitudinal study. In particular, the effects and complex interactions of interpersonal trauma- namely childhood trauma and domestic violence- psychological capacities- namely personality functioning, mentalizing and emotion regulation- and pandemic-related adversity on psychological distress in adults and families will be investigated. Furthermore, we aim to exploratorily investigate behavioral and cognitive consequences of the pandemic including vaccination status and attitude, media consumption, authoritarianism and conspiracy beliefs, as well as violence, maltreatment and posttraumatic growth during the pandemic.

## Research Questions

### Main Research Questions Cross-Sectional Data

(1) Is there a relationship between pandemic-related adversity and psychological distress?
  (1.1) Is pandemic-related adversity a predictor of psychological distress?

(2) Is there a relationship between childhood trauma, psychological capacities and pandemic-related adversity and psychological distress in adults and families?
  (2.1) Is the relationship between pandemic-related adversity and psychological distress moderated by childhood trauma?
  (2.2) Is the relationship between pandemic-related adversity and psychological distress mediated by psychological capacities (personality functioning, mentalizing, emotional experience and regulation)?
  (2.3) Is the relationship between pandemic-related adversity and psychological distress as well as infant/toddler regulatory problems mediated by parental psychological capacities (parental mentalizing, emotional experience and regulation)?
  (2.4) Is the relationship between pandemic-related adversity and psychological distress moderated by childhood trauma and mediated by psychological capacities (personality functioning, mentalizing, emotional experience and regulation)?

### Main Research Questions Longitudinal Data

(3) Is there a relationship between changes in pandemic-related adversity and psychological distress in course of the 1-year longitudinal study?
(4) Is there a relationship between childhood trauma, changes in psychological capacities and pandemic-related adversity and changes in psychological distress over time?
  (4.1) Is the relationship between changes in pandemic-related adversity and psychological distress predicted by childhood trauma?
  (4.2) Is the relationship between changes in pandemic-related adversity and psychological distress mediated by changes in psychological capacities (personality functioning, mentalizing, emotional experience and regulation)?
  (4.3) Is the relationship between changes in pandemic-related adversity and psychological distress as well as infant/toddler regulatory problem problems mediated by changes in parental psychological capacities (parental mentalizing, emotional experience and regulation)?
  (4.4) Is the relationship between changes in pandemic-related adversity and psychological distress predicted by childhood trauma and mediated by changes in psychological capacities (personality functioning, mentalizing, emotional experience and regulation)?

(5) Is there a relationship between changes in parental psychological capacities, pandemic-related adversity and violence and maltreatment during the pandemic?
  (5.1) Is the relationship between changes in pandemic-related adversity, violence and maltreatment during the pandemic mediated by changes in psychological capacities (parental reflective functioning, emotional experience and regulation)?

Additionally, further exploratory analyses with regard to the relationship between psychological capacities, pandemic-related adversity, psychological well-being and pandemic-related phenomena including conspiracy beliefs, media consumption, authoritarianism and vaccination status and attitudes will be conducted.

## Method

**FIGURE 1:**
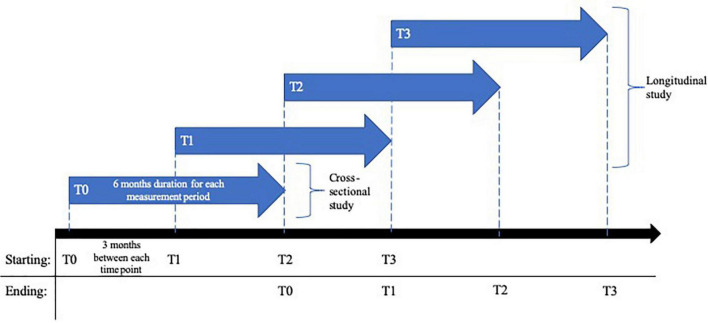
Design of the cross-sectional and 1-year longitudinal study on risk and protective factors of the psychosocial impact during the COVID-19 pandemic in Germany.

A cross-sectional and longitudinal study of the relationship between childhood trauma, personality functioning, mentalizing ability, pandemic-related stressors, and psychological distress will be conducted. The survey will be conducted online using the SoSci Survey platform. Ethic approval has been obtained from the local ethics board (Heidelberg University; AZ Tau 2020 3/1). The first measurement period (t0) began in August 2020 and continued through February 2021. After the first survey period t0, three additional follow-up surveys will be conducted at 3-months intervals. The first follow-up survey (t1) started in November 2020 and ended in May 2021. The second follow-up survey (t2) started in February 2021 and will end in August 2021. The third follow-up survey (t3) started in May 2021 and will end in November 2021. Figure 1 illustrates the study design with time measurement points.

### Sample

Adults over 18 years of age will be included. Aiming at a sample size of *N* = 800 at the last time point after 9 months and considering a drop-out rate of approx. 35% per time point (cf., Kujawa et al., 2020), appr. *N* = 3,000 adults (cross-sectional study) need to be included.

Participants will be recruited *via* the Institute for Psychosocial Prevention at Heidelberg University Hospital, *via* online and social media platforms (including Facebook, Instagram, Twitter, and Reddit), e-mails, and print flyers. Recruitment strategies are designed to reach a diverse population of German-speaking adults, with a focus on populations that may be particularly affected by the pandemic (including participants working in system-relevant occupations and families). Therefore, we particularly target respective groups on social media platforms (e.g., parent groups and forums) while we also recruit in more general groups/forums (e.g., COVID-19 related groups, sport clubs, and universities nationwide). Although our strategy aims at including a diverse population, participants’ characteristics will be analyzed for oversampling younger and better educated participants.

### Measures

Table 1 includes an overview of all measures used in the study and their respective assessment time point.

**TABLE 1:** Overview of PACE measures in cross-sectional and longitudinal surveys.

Construct	Measure	T0	T1	T2	T3
**Predictors, moderators and mediators**					
Sociodemographic characteristics	Questions about socio-economic factors, current living situation, financial and professional changes, place of residence (15 items)	x	x	x	x
Childhood trauma	Childhood Trauma Questionnaire -Short Form (CTQ; German version by Wingenfeld et al., 2010) (28 items)	x			
**Psychological capacities**					
Personality functioning	Personality Inventory for DSM-5 - Brief Form Plus (PID5BF +; Kerber et al., 2019) (34 items)	x	x	x	x
	Standardized Assessment of Severity of Personality Disorder – Short Form (SAS-PD; Olajide et al., 2018; German version by Zimmermann and Leising, 2015) (9 items)	x	x	x	x
Mentalizing abilities	Mentalization Scale [MentS; German version by Dimitrijević et al. (2017)] (28 items)	x	x	x	x
Parental reflective functioning	Parental Reflective Functioning Questionnaire: Subscale *Interest and Curiosity in Mental States* [German version by Ramsauer et al. (2014)] (5 items)	x	x	x	x
Emotional experience and regulation	Difficulties in Emotion Regulation Scale - Short Form (DERS-SF; German version by Ehring et al., 2008) (18 items)	x	x	x	x
	Positive and Negative Affect Schedule - Short Form [PANAS; German version by Breyer and Bluemke (2016)] (9 items)	x	x	x	x
	Child Abuse Potential Inventory: Two items *on anger and hostility* (CAPI; Deegener et al., 2009)	x	x	x	x
	Symptom-Checklist-90R-S: Subscales *Anger and hostility* (SCL-90R-S; Franke, 2014) (6 items)	x	x	x	x
**Pandemic-related adversity**					
Pandemic phases	A dimensional score specifying different phases of the lockdown	x	x	x	x
COVID-19 adversity scale	Questions about the burden of the social contact restrictions, changes in lifestyle, experience of lack of health care and family specific items, e.g., home schooling, partnership support (36 items)	x	x	x	x
**Psychological well-being**					
Psychological symptom severity	Patient Health Questionnaire [PHQ-ADS; German version by Kroenke et al. (2016)] (16 items)	x	x	x	x
Well-being	World Health Organization Well-Being Index (WHO-5; Brähler et al., 2007; Topp et al., 2015) (5 items)	x	x	x	x
Emotional and behavioral problems/regulatory difficulties in 0–3-year-old children	Baby-DIPS adapted questionnaire (BABY-DIPS; Popp et al., 2016)	x	x	x	x
	Questionnaire for Crying, Feeding, and Sleeping (QCFS; Gross et al., 2013) (17 items)	x	x	x	x
**Violence and maltreatment**					
Intimate partner violence during the pandemic	Partner Violence Screening adapted by including 3 items that capture sexual abuse and reasons to seek medical assistance related to violence (PVS; Nyberg et al., 2008) (5 items)		x	x	x
Child maltreatment during the pandemic	Kinder In Deutschland (children in Germany) 0–3 selected items (KID 0–3; Eickhorst et al., 2015) (7 items)		x	x	x
**Pandemic-related phenomena**					
Conspiracy beliefs	Conspiracy Mentality Questionnaire (CMQ; Bruder et al., 2013) (5 items)	x	x	x	x
Media consumption	Questions about media consumption in times of the pandemic (8 items)	x	x	x	x
Authoritarianism	Short Scale Authoritarianism (KSA-3; Beierlein et al., 2014) (9 items)	x	x	x	x
Vaccination status and attitude	Questions about vaccination status and intentions/attitude about vaccination (2 items)		x	x	x
Posttraumatic growth	Posttraumatic Growth Inventory (PTGI-SF; Cann et al., 2010; translated into German and back-translated as part of this study) (10 items)	x	x	x	x

#### Predictors, Moderators and Mediators

##### Sociodemographic Characteristics

At baseline general sociodemographic data using a standardized questionnaire including information about age, gender, educational and occupational background, and socio-economic situation will be collected and data about changes in sociodemographic background is collected at all follow-up timepoints.

##### Childhood Trauma

The childhood trauma questionnaire (CTQ; German version: Wingenfeld et al., 2010) is a 28-item retrospective self-report measure that is divided into five subscales to assess different aspects of maltreatment experiences in childhood: emotional abuse, physical abuse, sexual abuse, emotional neglect, and physical neglect. Each subscale consists of five statements, which can be rated with a five- point Likert Scale (1 = not at all; 5 = very often). Three additional items measure the individual’s tendency to minimize and deny. The subscale-scores range from 5 to 25 and the total score ranges from 25 to 125, with higher points indicating a greater extent of maltreatment. For each subscale specific cutoffs indicate the frequency of exposure. Strong psychometric properties have been demonstrated for the CTQ in clinical as well as community samples (Scher et al., 2001). The German version showed similar properties to the original version and demonstrated to be reliable and valid (Wingenfeld et al., 2010).

##### Psychological Capacities

###### Personality functioning

The PID-5 Brief Form + (PID5BF +; Kerber et al., 2019) is a brief self-report measure for assessing the six pathological trait domains described in AMPD criterion B and the ICD-11: negative affectivity, detachment, antagonism/dissociality, disinhibition, anankastia and psychoticism. Items are rated on a four-point scale ranging from 0 (“very false or often false”) to 3 (“very true or often true”). The internal consistency of the domain scores in three large samples was high (mean McDonald’s ω = 0.81), and domain scores were substantially positively correlated with each other.

The Standardized Assessment of Severity of Personality Disorder (SASPD; Olajide et al., 2018; German version: Zimmermann and Leising, 2015) is a brief measure to assess the severity of a potential personality disorder (PD) according to ICD-11 (Olajide et al., 2018). It consists of a total of nine items relating to different life domains and skills such as friendships, self-control, or compassion. Respondents can choose between four statements, which can then be recoded into a four-point scale according to the severity of a potential personality disorder (0 = Non-existent, 1 = Mild, 2 = Moderate and 3 = Severe). However, studies that addressed the validation of the SASPD found that the SASPD total score may be more useful as an indicator of a complex and heterogeneous mix of PD characteristics rather than actually representing severity (Rek et al., 2020). The SASPD nonetheless shows acceptable internal consistency in both clinical (ω = 0.78) and non-clinical (ω = 0.70) samples (Rek et al., 2020).

###### Mentalizing Abilities

The Mentalization Ability Assessment Questionnaire (MentS; Dimitrijević et al., 2018; German version: D-MentS by Dimitrijević et al., 2017) is a self-report questionnaire with 28 items to assess mentalization in clinical and healthy populations. Items are rated on a five-point scale (1 = “strongly disagree” to 5 “strongly agree”). The questionnaire consists of three subscales: MentS-A (10 items) captures mentalizing in relation to the other; MentS-S (8 items) captures mentalizing in relation to the self; and MentS-M (10 items) captures motivation to mentalize. A sum score maps the total mentalizing abilities score. Internal consistencies are acceptable in clinical and healthy populations and validity is adequately ensured (Dimitrijević et al., 2017).

###### Parental Reflective Functioning

We used the *interest and curiosity in mental states* scale of the German version of the Parental Reflective Functioning Questionnaire [PRFQ by Ramsauer et al. (2014); Luyten et al. (2017)] to assess parental reflective functioning. The scale consists of six items and assesses a parents’ proclivity to be interested in the child’s mental states. Items are rated on a seven-point Likert scale ranging from strongly disagree to strongly agree. The PRFQ reliably and validly assessed parental reflective functioning (Luyten et al., 2017; Anis et al., 2020).

###### Emotional Experience and Regulation

The Difficulties in Emotion Regulation Scale- Short Form (DERS-SF; German version: Ehring et al., 2008) is a 18-item self-report measure to assess emotional dysregulation across six domains: non-acceptance of emotional responses, lack of emotional awareness, limited access to ER strategies, lack of emotional clarity, difficulties engaging in goal-directed behavior, and impulse control difficulties when experiencing negative emotions. Participants rate the frequency with which the 18 statements apply to themselves by choosing from a five-point Likert-type scale. The items were recoded, with higher scores for each item and a higher overall score indicating more deficits in emotional dysregulation. The original version of the DERS showed high internal consistency (α = 0.93), good test-retest reliability, and adequate constructive and predictive validity (Gratz and Roemer, 2004). The German version of the DERS also demonstrated adequate to good internal consistencies within the original sample (0.76 < α < 0.87; Ehring et al., 2008).

The Positive and Negative Affect Schedule (PANAS; Janke and Glöckner-Rist, 2014) captures various feelings and emotions consisting of 20 items. With ten items each, the dimensions of positive and negative affect are captured on a 5-point Likert scale with response options “not at all” to “extremely.” Reliability assessment good Cronbach’s α values of 0.86 for the two dimensions of positive and negative affect (Janke and Glöckner-Rist, 2014).

Symptomatic anger was assessed using the anger/hostility subscale of the Symptom-Checklist-90 (SCL-90R-S; Franke, 2014). Six items are rated on a five-point Likert scale from 0 (*not at all*) to 4 (*extremely*) with higher scores indicating higher distress. The subscale has demonstrated sufficient internal consistency (α = 0.79; Franke, 2014).

In addition, two items from the German of the Child Abuse Potential Inventory (CAPI; Deegener et al., 2009) were utilized to assess general anger/hostility (English translation of the German version: “I am often angry inside”; “Many things in my life make me angry”). Items are rated on a four-point Likert scale. The CAPI is a valid and reliable inventory to determine the degree of stress of parents or other primary caregivers to assess the risk of child endangerment and abuse (Milner, 1994). Internal consistency (α = 0.91) is high (Deegener et al., 2009).

##### Pandemic-Related Adversity

###### Pandemic Phases

A dimensional score specifying different phases of the lockdown will be calculated. The score will be calculated based on the specific lockdown phase present at the assessment timepoint of each individual participant.

###### COVID-19 Adversity Scale

This summative score, developed as part of the project, is composed of items that are classified as stressful in the context of the COVID-19 pandemic. These include e.g., pandemic-related adversity experienced due to social contact restrictions, changes in life style (incl. activities, nutrition, exercise) and experience of lack of access to health care due to the pandemic. The COVID-19 adversity scale for families additionally covers experiences relevant to families and partnership support (incl. homeschooling, co-parenting). The conceptually derived scales (e.g., Benke et al., 2020; Kujawa et al., 2020; Rossi et al., 2020) will be empirically validated using confirmatory factor analyses.

#### Outcomes

##### Psychological Distress

###### Psychological Symptom Severity

The Patient Health Questionnaire (PHQ-D; German version: Löwe et al., 2008) represents a widely used, valid screening instrument based on the DSM-IV for the diagnosis of mental disorders. In order to assess psychological distress, the depression module (PHQ-9) with nine items and the module on generalized anxiety (GAD-7; English original version: Spitzer et al., 2006; German translation and validation: Löwe et al., 2008) with seven items are used in this study. Both modules are answered on a four-point scale (0 = “not at all” to 3 = “almost every day”) and are characterized by high validity, good internal consistencies (PHQ-9: α = 0.88; GAD-7: α = 0.89) and, especially for the depression module, by a high sensitivity to change (Gräfe et al., 2004; Löwe et al., 2008).

###### Regulatory Problems in Infants/Toddlers

Regulatory problems, which include excessive crying, sleep-onset, and night waking problems were assessed by parental report utilizing four screening items based on the structured diagnostic interview for regulatory problems in infancy (BABY-DIPS; Popp et al., 2016) and frequency and duration criteria of the Diagnostic Classification System DC:0-3R (Zero to Three, 2005). In addition, regulatory problems and related parental burden were assessed dimensionally utilizing 13 items from the Questionnaire for Crying, Feeding, and Sleeping (QCFS), which has been shown to be a valid measure for clinically significant regulatory disorders (Gross et al., 2013). Items are rated on a four-point Likert scale from *never/rarely* to *always/every day*. Higher scores signify more regulatory problems across all areas. Both instruments validly and reliably assess regulatory problems in infancy.

#### Further Secondary Outcomes

##### Psychological Well-being

###### Well-being

The German short version of the *World Health Organization Well-Being Index* (WHO-5; Brähler et al., 2007; Topp et al., 2015) consists of five items that refer to the well-being within the past 2 weeks. The response options are positively worded and refer to participants’ mood, calmness and relaxation, as well as perceived vitality, activity, restfulness of sleep and interest in things of daily living (Simon, 2017). The evaluation of the answers is recorded *via* a six-point Likert scale (0 = at no time; 5 = all the time). For scoring, the response scores of the five items are added together. The raw scores vary from 0 to 25 points, with a score of 0 being interpreted as the lowest psychological well-being and lowest quality of life, and a score of 25 being interpreted as the highest psychological well-being and highest quality of life. A score of <13 points is recommended as a cut-off criterion for poor psychological well-being. The internal consistencies of the test can be assessed as good with α = 0.88 (cf. α ≥ 0.89; Brähler et al., 2007; Döring and Bortz, 2016).

##### Violence and Maltreatment

###### Intimate Partner Violence During the Pandemic

The modified Partner Violence Screen (PVS, Nyberg et al., 2008) was used to assess exposure to partner violence during the last 12 months and particularly, since the beginning of the pandemic. The original PVS (Feldhaus et al., 1997) consists of three items that capture physical abuse and perceptions of safety. It has been translated to German and adapted by Nyberg et al. (2008) by including two items that capture sexual abuse and reasons to seek medical assistance related to violence. The measure has shown a sensitivity of 0.80 and a specificity of 0.78 (Nyberg et al., 2008). In this study, the PVS was modified in order to screen for violence against a partner of either gender and the item on medical assistance has been deleted due to the context of our study which does not provide crisis intervention.

###### Child Maltreatment During the Pandemic

We assessed child maltreatment and violence among parents with 7 items stemming from a nationwide survey on young families in Germany (KID 0-3; Eickhorst et al., 2015). The items cover instances of child abuse and neglect (e.g., “Has your child been shaken or pushed against a wall by an adult?”) and instances of partner violence (e.g., “Has one parent threatened the other parent seriously?”). All items are assessed dichotomously (yes/no). We asked participants to answer whether instance occurred before or since the beginning of the pandemic or both. The measure has been successfully used to assess child maltreatment in German young families.

##### Pandemic-Related Phenomena

###### Conspiracy Beliefs

The Conspiracy Mentality Questionnaire (CMQ) by Bruder et al. (2013) is an instrument that captures the conspiratorial mentality as a one-dimensional construct. On an eleven-point scale with a range from: 0% “certainly not” to 100% “certain” with a total of five-items, a generic conspiracy conviction is shown. The internal consistency of the questionnaire has been calculated and showed “good” results of Cronbach‘s Alpha for the German language version (α = 0.84). In addition, the model indices showed a very good fit (CFI > 0.95, RMSEA < 0.06), as well as factor loadings of >0.50. Furthermore, the test-retest-reliability in a 2-week interval was also satisfactory.

###### Media Consumption

Participants are asked about their media usage (e.g., type of newspapers, social media platforms, YouTube channels or podcasts) and indicate, which media they have used to obtain information about the Covid-19 pandemic.

###### Authoritarianism

The short scale authoritarianism (KSA-3) by Beierlein et al. (2014) is an economic instrument for assessing authoritarianism with its three sub-dimensions (aggression, submission and conventionalism). The scale comprises a total of nine items, with three items per sub-dimension. Those are rated on a five-point rating scale with a range from: (1) completely disagree to (5) completely agree. The factorial validity shows model indices of CFI = 0.962, RMSEA = 0.067, and SRMR = 0.043, which can be classified as satisfactory. Furthermore, factor loadings of all items on the scale (>0.50) and the three sub-dimensions on the general factor authoritarianism were shown. Using the McDonald ω coefficient, the reliability of the scales was determined, showing sufficient reliabilities of the three sub-dimensions of ω = 0.86 for authoritarian aggression, ω = 0.74 for authoritarian subservience, ω = 0.78 for conventionalism.

###### Posttraumatic Growth Inventory

Posttraumatic Growth Inventory (PTGI-SF; Cann et al., 2010) is the short form of the Posttraumatic Growth Inventory from Tedeschi and Calhoun (1996) and comprises 10 items about the positive outcomes of traumatic experiences. The five domains include New Possibilities, Relating to Others, Appreciation of Life, Spiritual Change and Personal Strength are measured with two items for each subscale. The calculation of the reliability has shown sufficient results (α = 0.90). Also, the Fit indices indicate a good fit (CFI = 0.965; RMSEA = 0.072). We adapted the instructions in order to assess posttraumatic growth related to the pandemic.

###### Vaccination Status and Attitude

We have included two questions about COVID-19 vaccination status of the participant and their close relatives as well as their motivation their general attitude toward COVID-19 vaccination.

### Statistical Analysis

Data within individual timepoints will be assessed for missing data and patterns of missingness. For missing at random data, we will conduct multiple imputation by chained equations (MICE) using the R package *mice* (Groothuis-Oudshoorn and Van Buuren, 2011). We will use predictive mean matching (Little, 1988) as imputation algorithm, since it is moderately robust when the empirical data deviate from distributional assumptions (Kleinke, 2017). Cross-sectional analysis: To answer research questions 1 to 2.4 on the association between childhood trauma, psychological capacities, pandemic-related adversity and psychological distress structural equation modeling will be applied for the cross-sectional data analyses. Longitudinal analysis: To answer the research questions 3 to 6.1 on the moderating and mediating effect of the named risk and protective factors regarding the relationship of pandemic-related adversity and psychological distress as well as potential associated pandemic-related phenomena, the data analyses of the longitudinal correlations are performed using multilevel modeling with measurement time points (level 1) nested in subjects (level 2). Here, changes in psychological distress at Level 1 is predicted by pandemic-related distress factors and linear time, as well as potential covariates (including age, gender). Psychological capacities are introduced as a mediator at Level 1. Analyses will be conducted using the Lavaan and semPlot-packages of the statistical software R.

## Discussion

This paper describes the study protocol of a cross-sectional and longitudinal study on risk and protective factors of mental health during the COVID-19 pandemic. To the best of the authors’ knowledge, this is the first study of its kind that investigates the moderating and mediating role of traumatic experiences and psychological capacities during the pandemic in a large community sample with a particular focus on families using a longitudinal design.

By expanding and testing the existing transdiagnostic models of childhood trauma and psychopathology (McLaughlin et al., 2020), and mentalizing (Luyten et al., 2020), this study will make an important contribution to a better understanding of risk and protective factors of mental health in the course of a worldwide pandemic crisis and the experience of related adverse events. Furthermore, the longitudinal examination of psychological capacities like personality functioning, mentalizing skills and emotion regulation in the relationship between pandemic-related stressors and psychological stress experience may provide valuable information for the development of pre- and intervention measures for potential psychological stress experience during and post the pandemic.

The strengths of this study include the broad and comprehensive recruitment approach, the investigation of theoretically grounded risk and protective factors of mental health during the COVID-19 crisis, in particular investigating a vulnerable population of families with young children.

Limitations of this study include a strategy that is primarily limited to online recruitment, which may potentially exclude individuals, who do not access these online platforms. Moreover, this sample is self-selected and will not be representative for the larger general population of Germany or Europe.

In addition, the survey is long and may attract only highly motivated individuals. Thus, our results will contribute to identifying risk and protective factors which, however, we cannot claim to be representative for the general population. In case of a large drop-out in the follow-up assessments, the sample will further suffer from self-selection processes. However, we will assess demographic characteristics in the follow-up surveys in order to accurately describe the sample in these aspects and will take on control measures for oversampling younger and better educated participants. Another limitation is the use of only self-report assessments, which may result in response biases and are limited to contents that are consciously processable. Finally, although we aim to assess pandemic-related adversity operationalized by including pandemic phases as well as a COVID-19 adversity scale retrospectively, the lack of pre-pandemic data limits our data validity regarding immediate effects caused by the pandemic-related restrictions and measures on mental health.

In light of the massive global psychological impact of the pandemic, this study with a strong focus on intra- and interpersonal psychological risk and protective factors will be of direct relevance to the evaluation of how pandemic measures affect vulnerable populations but also to the development of mental health and preventive services during and after the pandemic.

## Author Contributions

JV drafted the current manuscript. AB, LK, HW, JH, AG, and ST revised, corrected, and finally approved. All authors provided a substantial contribution to the conception and design of the work by developing the research question, study design, methodology, and agreed to be accountable for all aspects of the work in ensuring that questions related to the accuracy or integrity of any part of the work were appropriately investigated and resolved.

## Conflict of Interest

The authors declare that the research was conducted in the absence of any commercial or financial relationships that could be construed as a potential conflict of interest.

## Publisher’s Note

All claims expressed in this article are solely those of the authors and do not necessarily represent those of their affiliated organizations, or those of the publisher, the editors and the reviewers. Any product that may be evaluated in this article, or claim that may be made by its manufacturer, is not guaranteed or endorsed by the publisher.
